# Dopamine transporter single-photon emission computed tomography-derived radiomics signature for detecting Parkinson’s disease

**DOI:** 10.1186/s13550-022-00910-1

**Published:** 2022-06-27

**Authors:** Takuro Shiiba, Kazuki Takano, Akihiro Takaki, Shugo Suwazono

**Affiliations:** 1 Department of Molecular Imaging, School of Medical Sciences, Fujita Health University, 1-98, Dengakubo, Kutsukake-cho, Toyoake, Aichi 470-1192 Japan; 2 Department of Radiological Technology, Faculty of Fukuoka Medical Technology, Teikyo University, 6-22 Misakimachi, Omuta-shi, Fukuoka, 836-8505 Japan; 3 Department of Neurology and Center for Clinical Neuroscience, National Hospital Organization Okinawa National Hospital, 3-20-14 Ganeko, Ginowan, 901-2214 Okinawa Japan

**Keywords:** Parkinson’s disease, Dopamine transporter, SPECT, Radiomics signature, Texture, Radiomics

## Abstract

**Background:**

We hypothesised that the radiomics signature, which includes texture information of dopamine transporter single-photon emission computed tomography (DAT-SPECT) images for Parkinson’s disease (PD), may assist semi-quantitative indices. Herein, we constructed a radiomics signature using DAT-SPECT-derived radiomics features that effectively discriminated PD from healthy individuals and evaluated its classification performance.

**Results:**

We analysed 413 cases of both normal control (NC, *n* = 101) and PD (*n* = 312) groups from the Parkinson’s Progression Markers Initiative database. Data were divided into the training and two test datasets with different SPECT manufacturers. DAT-SPECT images were spatially normalised to the Montreal Neurologic Institute space. We calculated 930 radiomics features, including intensity- and texture-based features in the caudate, putamen, and pallidum volumes of interest. The striatum uptake ratios (SURs) of the caudate, putamen, and pallidum were also calculated as conventional semi-quantification indices. The least absolute shrinkage and selection operator was used for feature selection and construction of the radiomics signature. The four classification models were constructed using a radiomics signature and/or semi-quantitative indicator. Furthermore, we compared the classification performance of the semi-quantitative indicator alone and the combination with the radiomics signature for the classification models. The receiver operating characteristics (ROC) analysis was used to evaluate the classification performance. The classification performance of SUR_putamen_ was higher than that of other semi-quantitative indicators. The radiomics signature resulted in a slightly increased area under the ROC curve (AUC) compared to SUR_putamen_ in each test dataset. When combined with SUR_putamen_ and radiomics signature, all classification models showed slightly higher AUCs than that of SUR_putamen_ alone.

**Conclusion:**

We constructed a DAT-SPECT image-derived radiomics signature. Performance analysis showed that the current radiomics signature would be helpful for the diagnosis of PD and has the potential to provide robust diagnostic performance.

## Background

Parkinson’s disease (PD) is characterised by the degeneration of the nigrostriatal dopamine nerve and the appearance of inclusion bodies containing α-synuclein as the main component (i.e. Lewy bodies) [1–3]. The striatum to which dopamine neurones are projected is a nerve nucleus constituting the basal ganglia and comprises the caudate nucleus and putamen. Dopamine transporter (DAT) single-photon emission computed tomography (SPECT) contributes to the diagnosis of PD and Lewy body dementia by providing a SPECT image reflecting the DAT distribution density in the striatum. Generally, the evaluation of DAT-SPECT images is conducted via visual inspection, frequently supported by semi-quantitative ratios, such as the striatum uptake ratio (SUR) or specific binding ratio (SBR) [4–7]. In visual assessment, information regarding the asymmetry of the left and right striata and the spatial accumulation site of ^123^I-FP-CIT can be obtained [8–11].

A semi-quantitative analysis is hypothesised to eliminate subjectivity and experience differences among readers. Accurate semi-quantitative values may be helpful in the early diagnosis and prediction of the prognosis of PD [12].

Texture analysis [13] can quantitatively represent the heterogeneity of radiopharmaceutical uptake, such as a tumour, in a region of interest [14, 15]. In recent years, radiomics [16–19], which includes texture analysis, is expected to be used not only for diagnosis but also for predicting patient prognosis and determining treatment effects. Texture analysis has also been applied to DAT-SPECT, and texture features correlate with motor and cognitive functions and contribute to the prediction of motor functions [20]. Rahmin et al. [20] showed that Haralick’s texture features [21, 22] in the caudate nucleus correlated with the Unified Parkinson’s Disease Rating Scale and disease duration. Among a large number of texture features, only Haralick’s texture features by grey-level co-occurrence matrix were used in these studies. In recent years, many software that can easily calculate radiomics features, including morphology, histogram, and texture, have become widely used for study [23]. However, to our knowledge, constructing a radiomics signature from a wide range of candidate features of DAT-SPECT images and evaluating the classification performance of PD have not been reported. Although the conventional semi-quantitative indices have high classification accuracy [24, 25], they do not represent the homogeneity or heterogeneity of radiopharmaceutical distribution in the striatum. The image heterogeneity may become a disturbing factor, which is not well represented through semi-quantitative indices. Therefore, we hypothesised that the radiomics signature, which includes texture information from DAT-SPECT images, may assist semi-quantitative indices. In this study, we constructed a radiomics signature using the radiomics features derived from DAT-SPECT that effectively discriminated PD from healthy individuals and evaluated its classification performance.

## Materials and methods

### Participants

All data used in this study were obtained from the Parkinson’s Progression Markers Initiative (PPMI) database (www.ppmi-info.org/data). At enrolment in PPMI, PD subjects were required to be age 30 years or older, untreated with PD medications (levodopa, dopamine agonists, MAO-B inhibitors, or amantadine), within 2 years of diagnosis, Hoehn and Yahr < 3, and to have either at least two of resting tremor, bradykinesia, or rigidity (must have either resting tremor or bradykinesia) or a single asymmetric resting tremor or asymmetric bradykinesia. All PD subjects underwent dopamine transporter (DAT) imaging with ^123^I Ioflupane or vesicular monoamine transporter (VMAT-2) imaging with ^18^F AV133 (Australia only) and were only eligible if DAT or VMAT-2 imaging demonstrated dopaminergic deficit consistent with PD in addition to clinical features of the disease [26]. The dataset contained 790 pre-processed ^123^I-FP-CIT DAT-SPECT images acquired at the screening stage (accessed on 3 April 2021). This study selected a total of 462 subjects acquired with the two manufacturer’s SPECT systems [SIEMENS (dataset 1); 340 and GE (dataset 2); 122]. Dataset 1 excluded subjects whose diagnosis changed during follow-up (NC: 13, PD: 7), resulting in a final total of 320 subjects (NC: 81, PD: 239); dataset 2 included 122 subjects (NC: 20, PD: 102) with no subjects excluded. The remaining data were not used for the following reasons: the number was small when divided by manufacturer, and the manufacturer was unknown. Dataset 1 was divided into the training and test datasets at 7:3 so that the ratio of the NC and PD groups would be constant. Dataset 2 was used as the test dataset 2.

### Reconstruction and spatial normalisation of SPECT images

Reconstructed DAT-SPECT images were downloaded from the PPMI website. As per PPMI documentation, pre-processing steps were performed at the Institute for Neurodegenerative Disorders and included the following steps: SPECT imaging and reconstruction: SPECT imaging was acquired at each imaging centre as per the PPMI imaging protocol and sent to the institute for neurodegenerative disorders for processing. SPECT raw projection data were imported to a HERMES (Hermes Medical Solutions, Stockholm, Sweden) system for iterative reconstruction. Iterative reconstruction was performed without filtering. The reconstructed files were transferred to the PMOD (PMOD Technologies, Zurich, Switzerland) for subsequent processing. Attenuation correction ellipses were drawn on the images, and a Chang 0 attenuation correction was applied to images utilising a site-specific *μ* that was empirically derived from phantom data acquired during site initiation for the trial. Once attenuation correction was completed, a standard Gaussian three-dimensional (3D) 6.0 mm filter was applied.

Then, the DAT-SPECT images were spatially normalised to Montreal Neurologic Institute (MNI) space using statistical parametric mapping (SPM12, Wellcome Trust Centre for Neuroimaging, London, UK) in MATLAB R2021a (version 9.10, The MathWorks, Inc. Massachusetts, USA). DAT-SPECT images were spatially normalised to the MNI-based template of ^123^I-FP-CIT [27, 28] using the old normalise function under identical conditions. After spatial normalisation, the radiological technologist with 13-year clinical experience visually assessed for misalignment between DAT-SPECT and the template. Visual assessment of spatial normalisation checked for apparent misalignment in the striatum and whole brain. The pre-processed images were saved in the Neuroimaging Informatics Technology Initiative format using 91 × 109 × 91 isotropic voxels of 2 mm.

### Calculation of radiomics features and semi-quantitative indicators

**Fig. 1:**
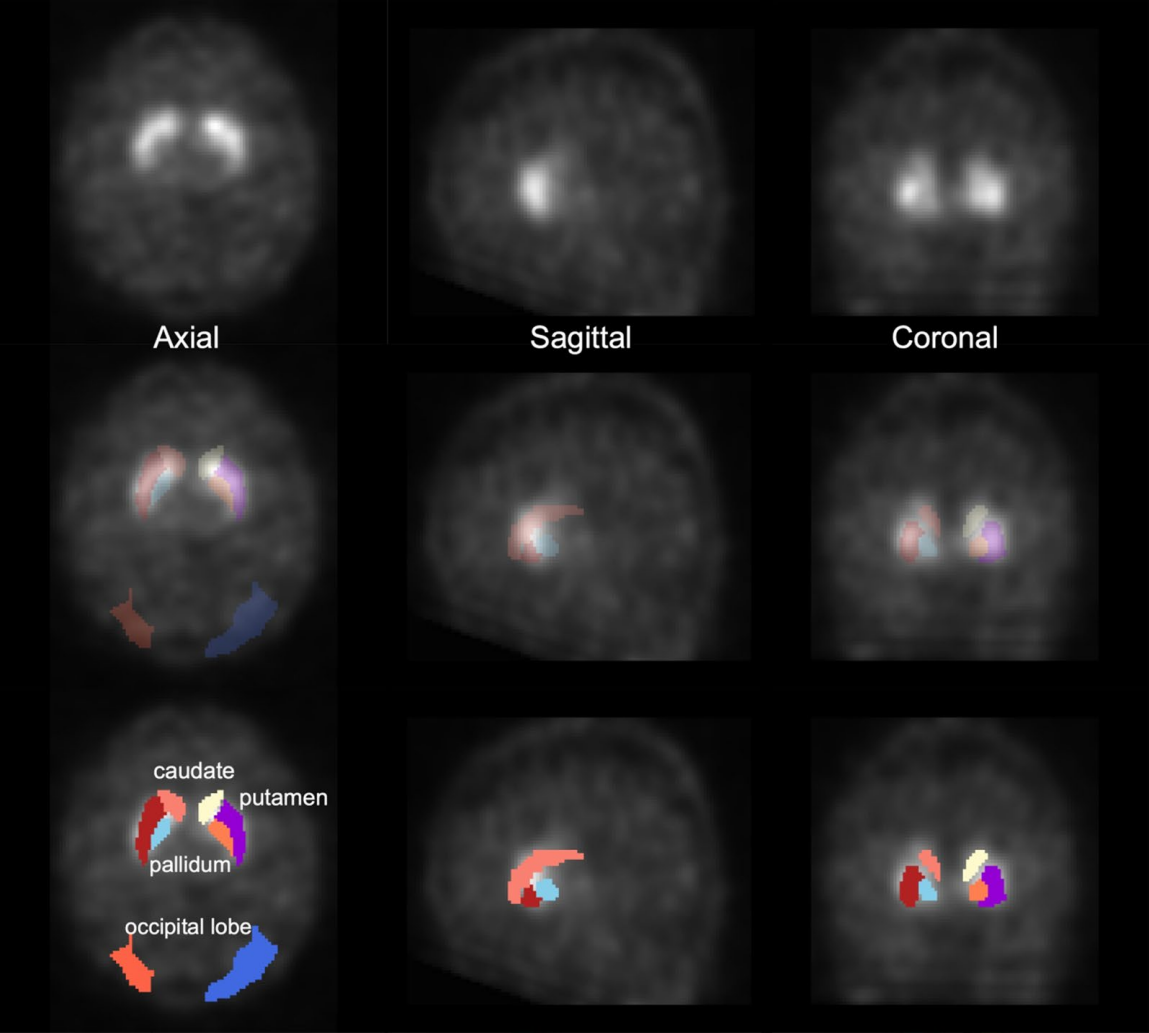
An example of settings of a voxel of interests for calculating radiomics features and semi-quantitative indices. The upper row indicates after the spatial normalisation dopamine transporter single-photon emission computed tomography, and the middle and lower rows indicate fused image. The coloured area of the middle and lower rows indicates the striatum and background (occipital lobe)

The automated anatomical labelling atlas (AAL) 3 [29] volume of interest (VOI) template was used to calculate the radiomics features. The feature calculation VOIs were the caudate nucleus, putamen, and pallidum (Fig. 1). Radiomics features were calculated using Standardized Environment for Radiomics Analysis (SERA) [30–32] and worked on MATLAB. One hundred and eighty-six image biomarker standardisation initiative-standardised features [23] were calculated using SERA, including 50 first-order features (statistical, histogram, and intensity histogram features) and higher-order136 3D features (Table 1). A total of 558 radiomics features were calculated for the caudate, putamen, and pallidum VOIs. We also calculated the ratio of the caudate to the putamen or pallidum of radiomics features. All radiomics features were averaged in the bilateral striatum part. These totalled 930 radiomics features. Furthermore, the SUR of the caudate nucleus (SUR_caudate_), putamen (SUR_putamen_), and pallidum (SUR_pallidum_) was calculated as conventional semi-quantification indices. The SUR was calculated using the following formula [33]:

$${\text{SUR}}\left( \% \right) = \frac{{C_{{{\text{striatum}}}} - C_{{{\text{background}}}} }}{{C_{{{\text{background}}}} }} \times 100$$

where *C*_striatum_ is the average count of the caudate nucleus, putamen, or pallidum, and *C*_background_ is the average count of the occipital lobe. In addition, the ratios of the caudate to the putamen or pallidum (CR_putamen_, CR_pallidum_) were calculated. All the semi-quantitative indices were averaged in the bilateral striatum and compared between the NC and PD groups, and receiver operating characteristic (ROC) [34] analysis was performed.

**Table 1:** Number of radiomics features per region and their family names

Feature family	Number of features
Local intensity	2
Intensity-based statistics	18
Intensity histogram	23
Intensity-volume histogram	7
GLCM	50
GLRLM	32
GLZSM	16
GLDZM	16
NGTDM	5
NGLDM	17
Total	186

*GLCM* grey-level co-occurrence matrix, *GLRLM* grey-level run-length matrix, *GLZSM* grey-level zone size matrix, *GLDZM* grey-level distance zone matrix, *NGTDM* neighbourhood grey tone difference matrix, *NGLDM* neighbourhood grey-level dependence matrix

### Radiomics feature selection and signature construction

The least absolute shrinkage and selection operator (LASSO) [35] function in MATLAB was used to select effective features from the radiomics features. Multi-collinearity of features was not considered in this study because LASSO can feature selection with suppressed multi-collinearity [36]. All radiomics features were z-scored to mean 0 and standard deviation 1.0 before being inputted to LASSO. LASSO permits the estimation and selection of explanatory variables [37, 38], that is, radiomics features with nonzero coefficients. For the selection of radiomics features using LASSO, a tenfold cross-validation test was conducted using the training set. Furthermore, the linear combination sum of five radiomics features with nonzero coefficients was used as the radiomics signature. We compared the classification performance of the radiomics signature and semi-quantitative indicator that showed the highest classification performance.

### Classification model construction with radiomics signature and semi-quantitative indicator

The classification models for the NC and PD groups were constructed using the radiomics signature and/or semi-quantitative indicator. The four classifiers used were support vector machine (SVM), k-nearest neighbour (KNN), linear discriminant analysis (LDA), and decision tree. The main parameters of each classifier were as follows: SVM (BoxConstraint = 1, KernelScale = 1, KernelFunction = polynomial [order = 3]), KNN (NumNeighbours = 1, Distance = Minkowski, Exponent = 2), LDA (Gamma = 0), and decision tree (MinLeafSize = 1, MinParentSize = 10). The features used were radiomics signature alone, semi-quantitative indicator alone, and both. The training set was used to train the classifier, and the performance of each classification model was evaluated using each test dataset. Classification performance was evaluated using the area under the ROC curve (AUC).

### Statistical analyses

The radiomics signature and SURs in the NC and PD groups were tested for significant differences using the Wilcoxon rank-sum test. ROC analysis was performed using semi-quantitative indicators and radiomics signature. We used the DeLong [39] test to examine the differences in AUCs, and for multiple comparisons, the Bonferroni correction was performed. The sensitivity, specificity, and accuracy of semi-quantitative indices and radiomics signature were calculated using the optimal cut-off values determined based on ROC analysis. The optimal cut-off values for radiomics signature and semi-quantitative indices were calculated using the training dataset. At the same time, sensitivity, specificity, and accuracy were assessed using test datasets 1 and 2.

Differences were considered statistically significant at *P* < 0.05. All statistical analyses were performed using RStudio (version 1.4.1106).

## Results

Table 2 shows the characteristics of the subjects in this study. In dataset 1, no cases were excluded due to failure of spatial normalisation, whereas in dataset 2, 28 subjects of PD were excluded due to failure of spatial normalisation, resulting in 93 subjects (NC: 20, PD 73). The number of subjects included 320 subjects for dataset 1 and 93 subjects for dataset 2.

**Table 2:** Characteristics of subjects

Characteristics	Dataset 1 (SIEMENS)		Dataset 2 (GE)	
	NC	PD	NC	PD
Number of subjects	81	239	20	73
Male/female	57/24	151/88	13/7	53/20
Age	61.2 ± 9.5	59.8 ± 11	60.3 ± 13	60.8 ± 10
Age of diagnosis	NA	60.7 ± 9.5	NA	59.0 ± 11
Hoehn and Yahr stage	0.01 ± 0.1	1.58 ± 0.5	0.00 ± 0.0	1.64 ± 0.5
MDS-UPDRS III	1.0 ± 1.9	21.8 ± 9.3	0.89 ± 2.2	17.0 ± 9.9
MoCA	28.2 ± 1.1	27.3 ± 2.3	28.6 ± 1.2	27.1 ± 3.3

*MDS-UPDRS* movement disorder society-unified Parkinson’s disease rating scale, *MoCA* Montreal Cognitive Assessment, *NC* normal control, *PD* Parkinson’s disease, *NA* not applicable

**Fig. 2:**
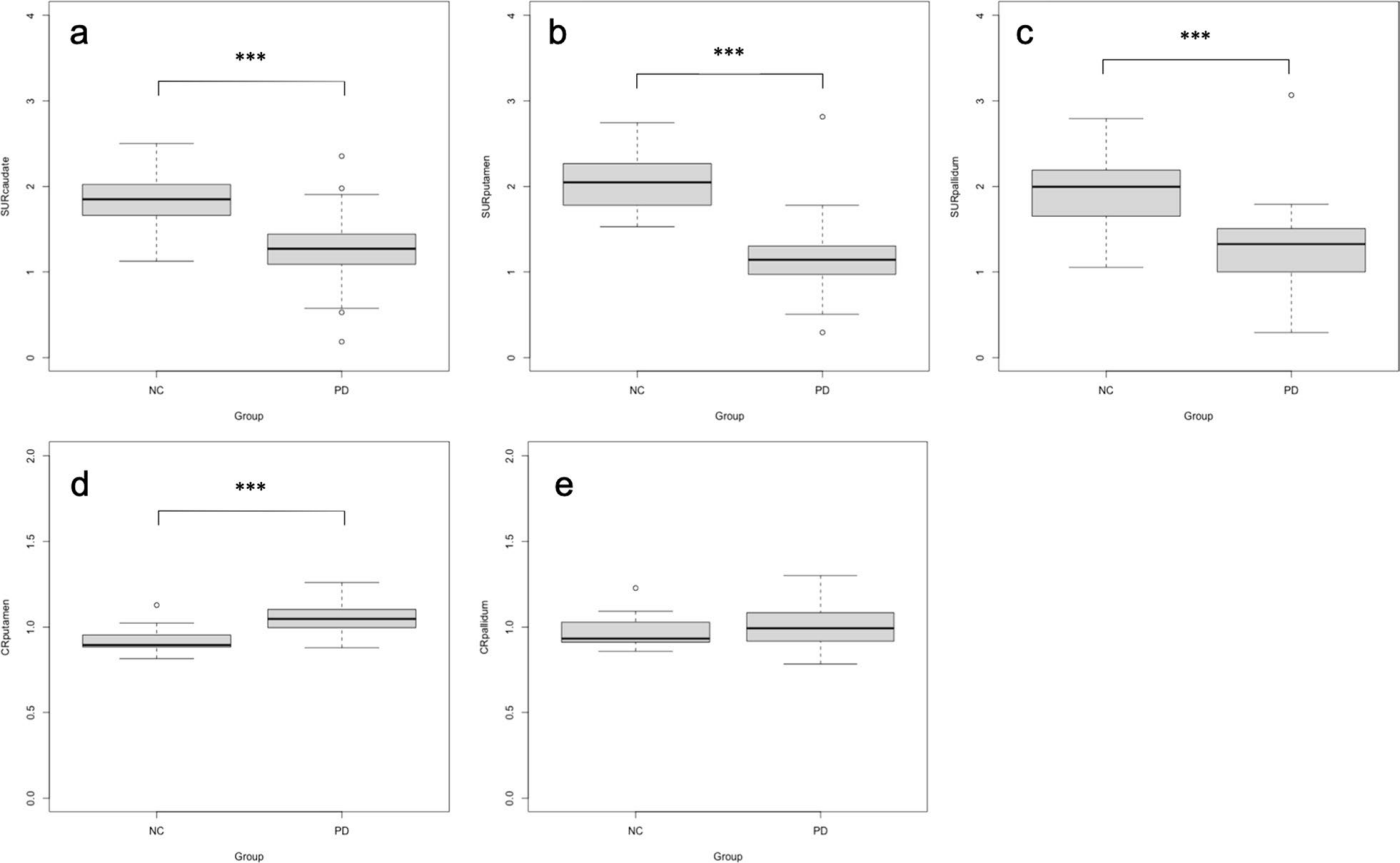
Striatum uptake ratio (SUR) and caudate ratio (CR) between the normal control and Parkinson’s disease for test dataset 1. Box-and-whisker plots indicate the semi-quantitative indices distribution. **a** SUR_caudate_, **b** SUR_putamen_, **c** SUR_pallidum_; **d,** CR_putamen_, **e** CR_pallidum_. ^***^P < 0.001

**Fig. 3:**
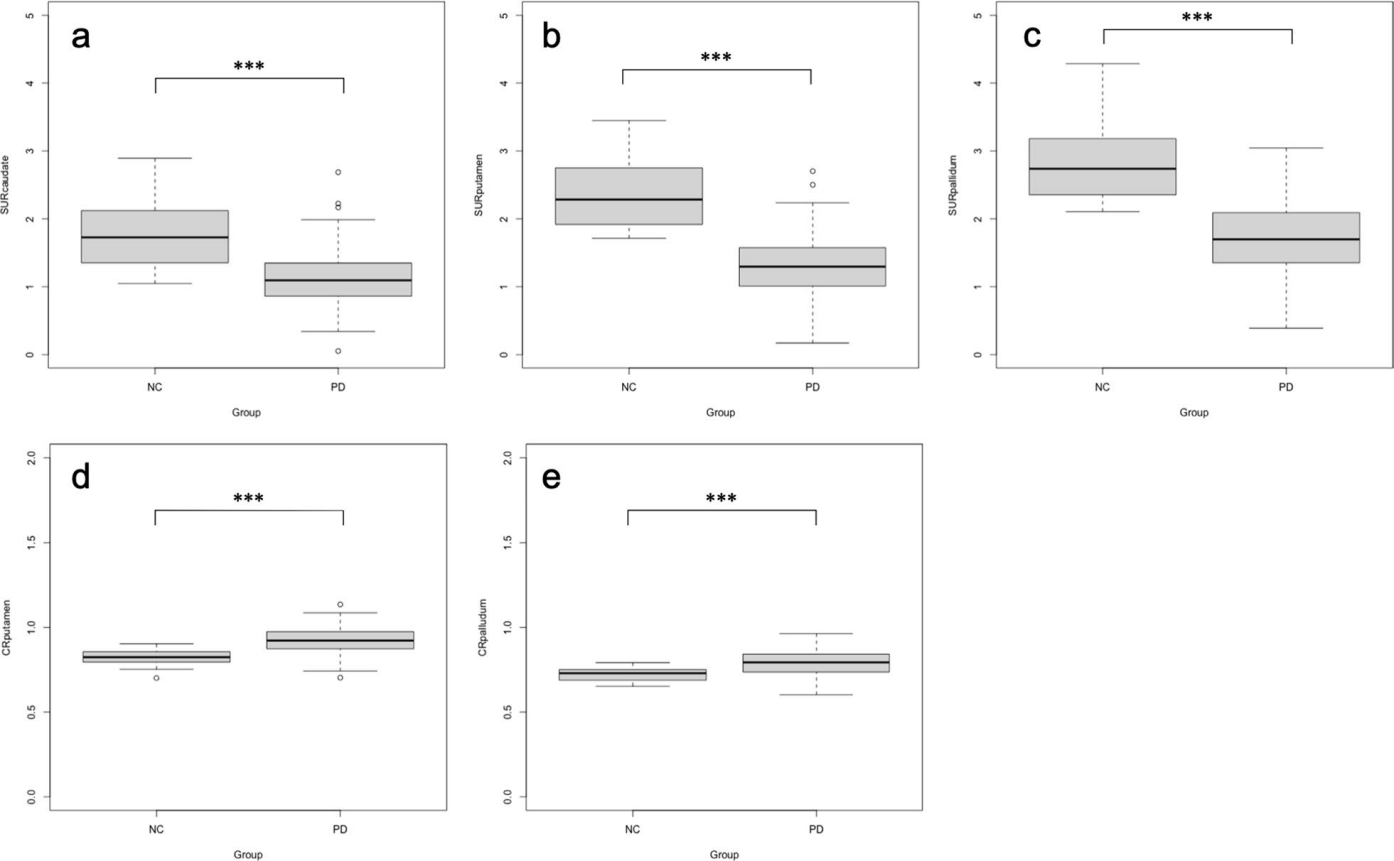
Striatum uptake ratio (SUR) and caudate ratio (CR) between the normal control and Parkinson’s disease for test dataset 2. Box-and-whisker plots indicate the semi-quantitative indices distribution. **a** SUR_caudate_, **b** SUR_putamen_, **c** SUR_pallidum_; **d,** CR_putamen_, **e** CR_pallidum_. ^***^P < 0.001

Figures 2 and 3 show the distribution of semi-quantitative indices for test datasets 1 and 2. There was a significant difference between the NC and PD groups in all of the SURs (*P* < 0.001). Caudate-to-putamen or pallidum ratios showed significant differences (*P* < 0.001) between NC and PD, except for CR_pallidum_ (*P* = 0.064) in test dataset 1.

**Fig. 4:**
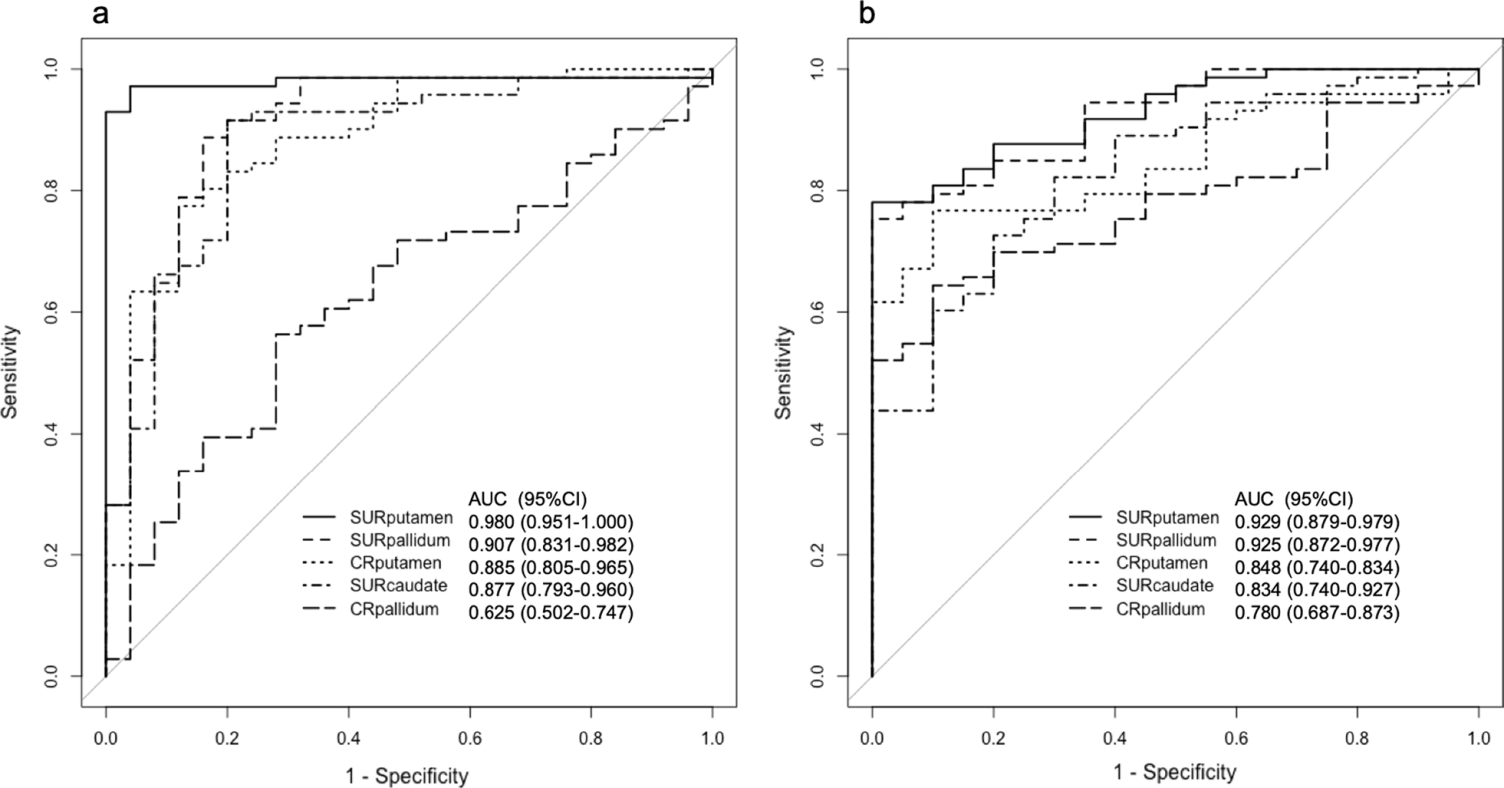
Receiver operating curves for semi-quantitative indices. **a** The AUCs and 95% confidence interval (CI) for the test dataset 1of semi-quantitative indices in each region were SUR_putamen_ (0.980, 0.951–1.000), SUR_pallidum_ (0.907, 0.831–0.982), CR_putamen_ (0.885, 0.805–0.965), SUR_caudate_ (0.877, 0.793–0.960), and CR_pallidum_ (0.625, 0.502–0.747). There was a significant difference between SUR_putamen_ and other semi-quantitative indices (*P* < 0.001). **b** The AUCs and 95% confidence interval (CI) for the test dataset 2 of semi-quantitative indices in each region were SUR_putamen_ (0.929, 0.879–0.979), SUR_pallidum_ (0.925, 0.872–0.977), CR_putamen_ (0.848, 0.740–0.834), SUR_caudate_ (0.834, 0.740–0.927), and CR_pallidum_ (0.780, 0.687–0.873). There was a significant difference between SUR_putamen_ and SUR_caudate_, (*P* < 0.01), or CR_pallidum_ (*P* < 0.05). *AUC* area under the curve, *CI* confidence interval, *SUR* striatum uptake ratio, *CR* caudate ratio

Figure 4 shows the ROC curves of the semi-quantitative indices for each test dataset. The AUCs and 95% confidence interval (CI) of SURs and CRs for test dataset 1 were, in order from highest to lowest, as follows: SUR_putamen_ (0.980, 0.951–1.000), SUR_pallidum_ (0.907, 0.831–0.982), CR_putamen_ (0.885, 0.805–0.965), SUR_caudate_ (0.877, 0.793–0.960), and CR_pallidum_ (0.625, 0.502–0.747). Similarly, for test dataset 2 as follows: SUR_putamen_ (0.929, 0.879–0.979), SUR_pallidum_ (0.925, 0.872–0.977), CR_putamen_ (0.848, 0.749–0.834), SUR_caudate_ (0.834, 0.740–0.927), and CR_pallidum_ (0.780, 0.687–0.873). There was a significant difference between SUR_putamen_ and other SURs or CRs (*P* < 0.05) for test dataset 1. For test dataset 2, there was a significant difference between SUR_putamen_ and two indices (SUR_caudate_ and CR_pallidum_) (*P* < 0.05).

Table 3 shows the radiomics features and coefficients selected by LASSO in the training set. The lambda value was set to 0.0967, and five radiomics features were selected. The selected radiomics features included four putamen features and CR_pallidum_. Coefficients and radiomics features were used to construct the radiomics signature:

**Table 3:** Radiomics features and coefficients selected using LASSO

Feature family	Feature name (tag name)	Region	Coefficients
Intensity histogram	Median (ih_median)	Putamen	− 0.00863
GLDZM (3D)	Zone distance non-uniformity (dzm_zdnu_3D)	Putamen	− 0.18100
NGLDM (3D)	Dependence count non-uniformity normalised (ngl_dcnu_norm_3D)	Putamen	− 0.02485
NGLDM (3D)	Dependence count non-uniformity normalised (ngl_dcnu_norm_3D)	Putamen	− 0.00001
GLSZM (3D)	Large zone low grey-level emphasis (szm_lzlge_3D)	Caudate/pallidum	− 0.05259

*GLDZM* grey-level distance zone matrix, *NGLDM* neighbourhood grey-level dependence matrix, GL*SZM* grey-level size zone matrix

Radiomics signature =  − 0.00863 × ih_median_putamen_ − 0.18100 × dzm_zdnu_3D_putamen_ − 0.02485 × ngl_dcnu_3D_putamen_ − 0.00001 × ngl_dcnu_norm_3D_putamen_ − 0.05259 × szm_lzlge_3D_CRpallidum_.

**Fig. 5:**
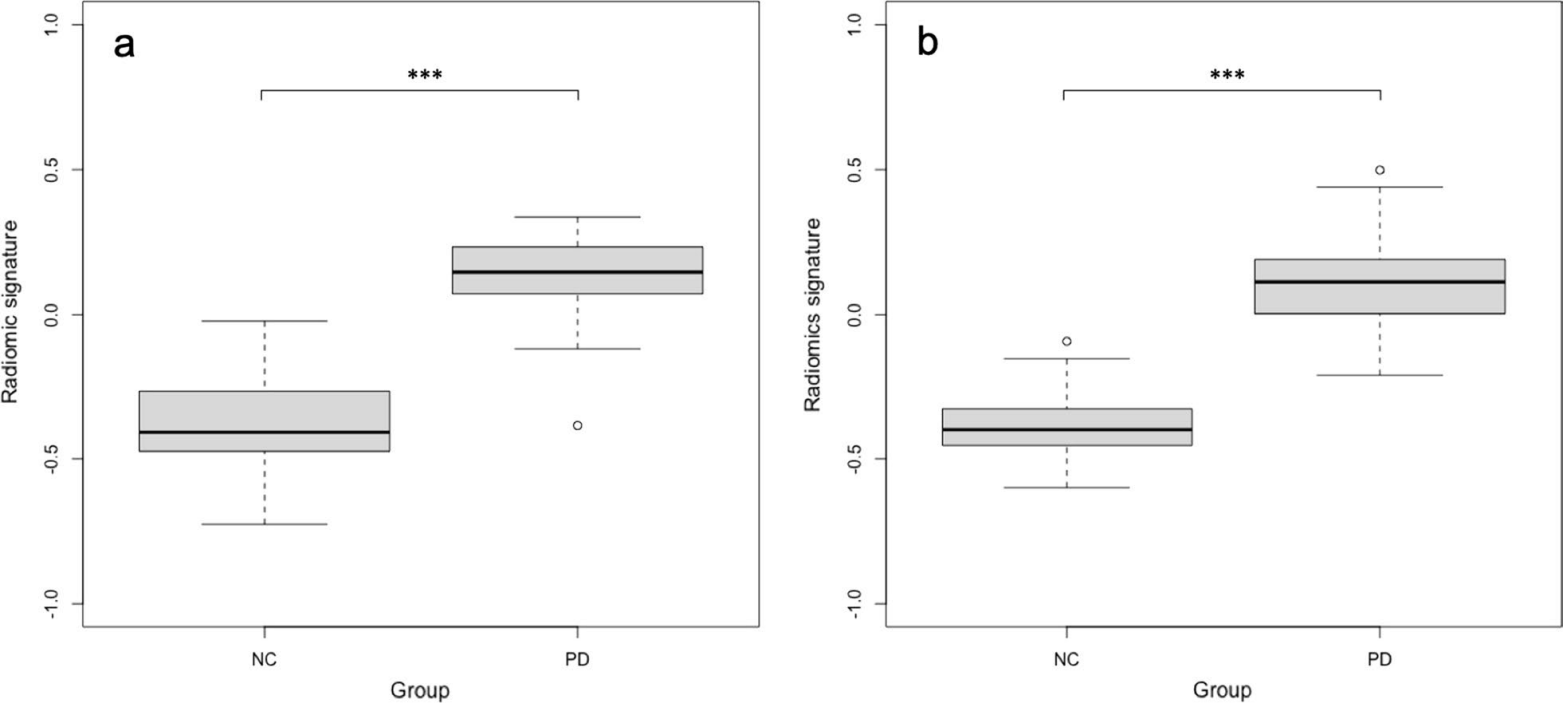
Comparison of radiomics signature between the normal control and Parkinson’s disease. Box-and-whisker plots indicate the radiomics signature distribution. **a** Test dataset 1, **b** test dataset 2. ^***^P < 0.001

Figure 5 shows the distribution of radiomics signatures between the NC and PD groups. There was a significant difference between the NC and PD groups (*P* < 0.001) for test datasets 1 and 2.

**Fig. 6:**
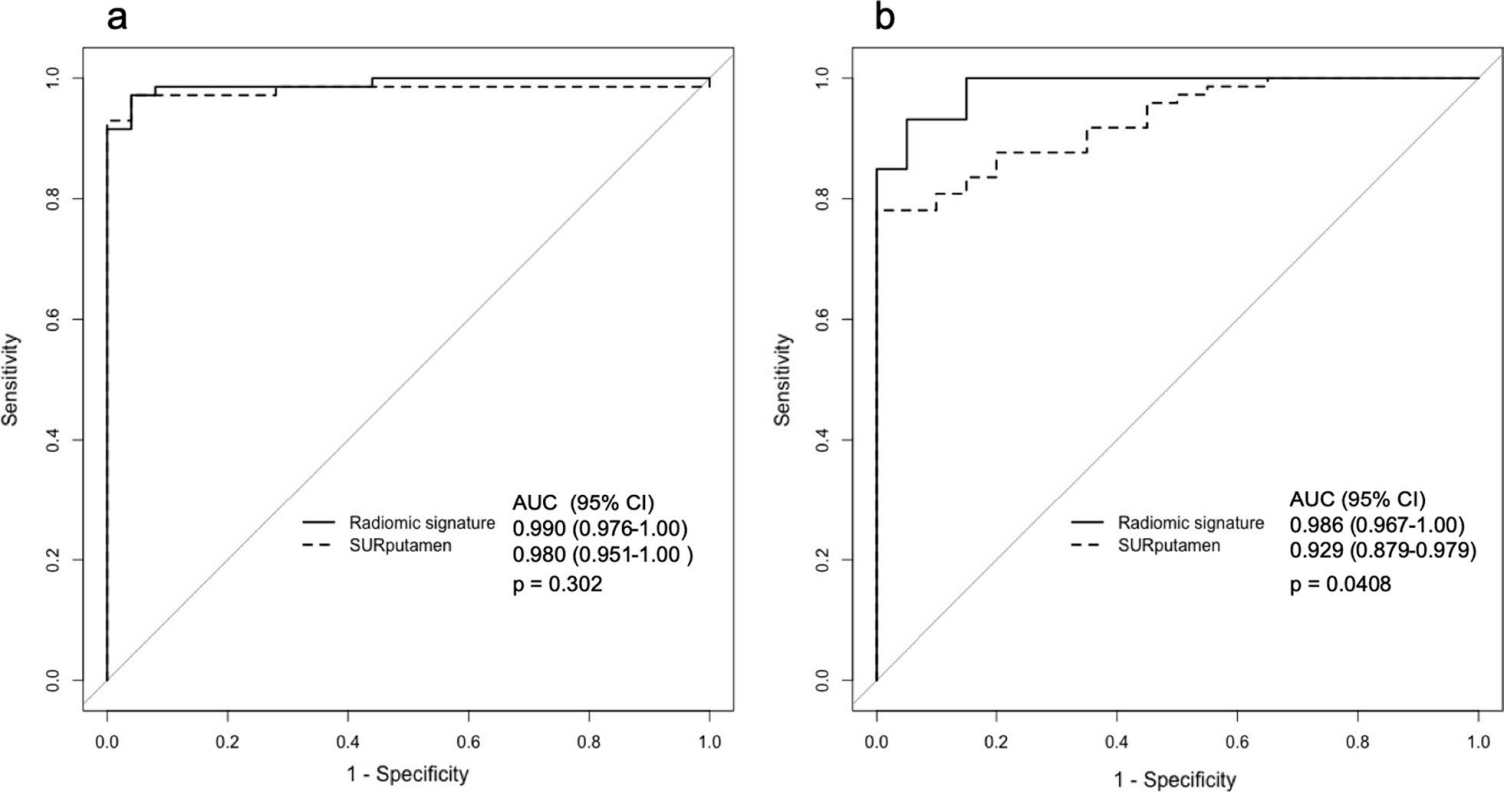
Comparison of receiver operating curves between radiomics signature and striatum uptake ratio of the putamen. **a** The AUCs for the test dataset 1of the radiomics signature and SUR_putamen_ were 0.990 (95% CI, 0.976–1.00) and 0.980 (95% CI, 0.951–1.00), respectively (*P* = 0.302). **b** The AUCs for the test dataset 2 of the radiomics signature and SUR_putamen_ were 0.986 (95% CI, 0.967–1.00) and 0.929 (95% CI, 0.879–0.979), respectively (*P* = 0.041). *AUC* area under the curve, *CI* confidence interval, *SUR* striatum uptake ratio

A comparison of the ROC curves for the radiomics signature and SUR_putamen_ is shown in Fig. 6. In the test dataset 1, the AUCs of the radiomics signature and SUR_putamen_ were 0.990 (95% CI, 0.976–1.00) and 0.980 (95% CI, 0.951–1.00), respectively (*P* = 0.302). In the test dataset 2, the AUCs of the radiomics signature and SUR_putamen_ were 0.986 (95% CI, 0.967–1.00) and 0.929 (95% CI, 0.879–0.979), respectively (*P* = 0.041).

Table 4 shows the classification accuracy of the radiomics signature and SUR_putamen_. The accuracy, sensitivity, and specificity of the radiomics signature and SUR_putamen_ were 95.8%, 98.6%, and 88.0% and 95.8%, 97.2%, and 92.0% and 96.8%, 100%, and 85.0% and 82.8%, 78.1%, and 100% for the test datasets 1 and 2, respectively.

**Table 4:** Classification accuracy of radiomics signature and SUR_putamen_ for the test datasets

	Test dataset 1		Test dataset 2	
	Radiomics signature	SUR_putamen_	Radiomics signature	SUR_putamen_
Accuracy (%)	95.8	95.8	96.8	82.8
Sensitivity (%)	98.6	97.2	100	78.1
Specificity (%)	88.0	92.0	85.0	100

*SUR* striatum uptake ratio

Tables 5 and 6 show the AUCs of each classification model when radiomics signature alone, SUR_putamen_ alone, and both features were combined. There were no significant differences in the AUCs between SUR_putamen_ alone and in combination with SUR_putamen_ and radiomics signature for test dataset 1. However, the AUC was better in all models when combined with SUR_putamen_ and radiomics signature compared to SUR_putamen_ alone.

**Table 5:** Classification performance of various classification models using radiomics signature and SUR_putamen_ for test dataset 1

Classifier	Features AUC (95% CI)			*P* value (SUR_putamen_ vs. combination)
	Radiomics signature	SUR_putamen_	Combination	
SVM	0.983 (0.943–0.996)	0.983 (0.944–0.996)	0.991 (0.967–0.999)	0.333
KNN	0.930 (0.835–0.979)	0.918 (0.817–0.972)	0.950 (0.866–0.991)	0.261
LDA	0.990 (0.956–0.999)	0.980 (0.910–0.997)	0.987 (0.931–1.000)	0.167
Decision tree	0.970 (0.877–0.999)	0.965 (0.909–0.988)	0.970 (0.879–0.998)	0.825

*SVM* support vector machine*, KNN* k-nearest neighbour*, LDA* linear discriminant analysis*, SUR* striatum uptake ratio, *AUC* area under the curve, *CI* confidence interval

**Table 6:** Classification performance of various classification models using radiomics signature and SUR_putamen_ for test dataset 2

Classifier	Features AUC (95% CI)			*P* value (SUR_putamen_ vs. combination)
	Radiomics signature	SUR_putamen_	Combination	
SVM	0.977 (0.934–0.994)	0.921 (0.847–0.963)	0.940 (0.746–0.995)	0.755
KNN	0.896 (0.768–0.998)	0.857 (0.729–0.941)	0.997 (0.986–1.000)	0.006
LDA	0.986 (0.947–0.998)	0.929 (0.858–0.967)	0.996 (0.961–1.000)	0.009
Decision tree	0.973 (0.929–0.990)	0.928 (0.858–0.966)	0.952 (0.909–0.985)	0.300

*SVM* support vector machine*, KNN* k-nearest neighbour*, LDA* linear discriminant analysis*, SUR* striatum uptake ratio, *AUC* area under the curve, *CI* confidence interval

A similar trend to test dataset 1 was observed in test dataset 2. Radiomics signature in combination with SURputamen improved AUC for KNN and LDA models.

## Discussion

In this study, we constructed and evaluated the potential of a radiomics signature derived from DAT-SPECT images to classify the NC and PD groups.

The main findings of this study are as follows. First, radiomics signature may have a similar or slightly higher classification performance than semi-quantitative indicators. Second, the combination of radiomics signature and semi-quantitative indicator as features for the classification models would improve the classification performance compared to that of the semi-quantitative indicator alone.

SUR_putamen_ showed the highest classification performance among the semi-quantitative indices for each region obtained from spatially normalised DAT-SPECT images. It is well known that ^123^I-FP-CIT decline began in the caudal putamen loss in patients with PD [40]. Therefore, SUR_putamen_ reflected the difference in radiopharmaceutical accumulation in the putamen between the NC and PD groups and showed high classification performance. The high classification performance of the semi-quantitative indicator of the putamen is consistent with those of several previous studies [41–43].

For radiomics feature selection, the most common region to which the eight features selected by LASSO belonged to the putamen, followed by the pallidum. This is because radiopharmaceuticals accumulate less from the putamen in PD, similar to the above. Radiomics features reflect the heterogeneity of radiopharmaceutical accumulations in VOIs. GLDZM (zone distance non-uniformity; tag name, dzm_zdnu_3D_putamen_), which had the most significant coefficient, is a matrix that shows how far the connected regions with the same concentration value are from the edge of the region of interest. The dzm_zdnu_3D measures the distribution of zone counts over the different zone distances and is low when the zone counts are equally distributed along with the zone distances. In the putamen region, dzm_zdnu_3D_putamen_ in the PD group was significantly lower than in the PD group (data not shown). This result indicates that the number of connected zones of PD was lower than that of the NC group. Comparing the histogram features in the putamen region of NC and PD in dataset 1, uniformity (0.03 vs 0.04, *p* < 0.001), kurtosis (−0.83 vs 0.24, *p* < 0.001), and skewness (0.21 vs 0.82, *p* < 0.001) of PD were higher than those of NC. These results indicate that voxel values in the putamen region of NC were widely distributed, whereas they tend to be biased towards lower voxel values in PD. In other words, the number of connected voxels per connected region was higher in PD because the voxel values were similar to each neighbouring voxel [the number of connected regions (= zone counts) was lower]. On the other hand, NCs are more likely to have different neighbouring voxel values, which means they may have fewer connected voxels per connected region and a larger number of connected regions than PD. In PD, the loss of dopamine transporters progresses from the posterior to the anterior of the putamen. The lower dzm_dcnu_3D in putamen for PD suggests large areas of reduced dopamine transporter. Based on the results of the two test datasets for radiomics signature, texture information of the putamen can be a robust and powerful indicator for the differentiation of PD.

The radiomics signature showed a similar or slightly higher classification performance between the PD and NC groups than that of SUR_putamen_. Furthermore, when the various classification models were constructed using both the radiomics signature and SUR_putamen_ as features, the classification performance was better than that of SUR_putamen_ alone. This result suggests that the radiomics signature provides robust texture information to supplement the semi-quantitative indicators. Iwabuchi et al. [44] reported that the combined diagnostic accuracy of the three types of indices, SBR, putamen-to-caudate ratio (PCR), and fractal dimension (or asymmetry index), used for SVM improves diagnostic accuracy. Generally, semi-quantitative indicators assess the quantity of radiopharmaceuticals, and their spatial distribution depends on visual assessment. Adding an indicator for the radiopharmaceutical spatial distribution (e.g. texture information, PCR, fractal dimension) to the semi-quantitative indicator would improve the diagnostic accuracy. We believe that the combination of the semi-quantitative indicator and radiomics signature would lead to the development of highly accurate automatic diagnosis or diagnostic assistant models. On the other hand, constructing a radiomics signature is more complicated and time-consuming than conventional semi-quantitative indicators such as SBR. The meaning indicated by the radiomics signature might be difficult to understand for physicians.

This study investigated the robustness of the radiomics signature by using two test datasets. The radiomics signature showed a high classification performance in each test dataset, which could be a robust indicator for PD and NC classification; SUR_putamen_ also showed a high classification performance, which was slightly lower in test dataset 2. The differences in image quality due to SPECT system were reflected in the SURs [45]. Furthermore, the difference in image quality may also affect the accuracy of spatial normalisation. In dataset 2, 28 cases of spatial normalisation failed. This failure was due to the lower prefrontal cortex and cerebellar areas omitting from the field of view. These SPECT images were taken at the same facility and may be an issue of imaging technique. In this study, VOI settings based on the AAL label images were applied to DAT-SPECT images after spatial normalisation. Buchert et al. [46] reported that the diagnostic performance of the caudate SBR was lower than that of putamen when using the VOI of AAL. Nonokuma et al. [47] used an MRI-based ROI similar to the AAL VOI but failed to accurate radioactivity in the caudate nucleus. They described that the tissue mixture effect due to the dilated anterior horn of the lateral ventricle might decrease the radioactivity in the caudate nucleus and shift the peak caudally. Similarly, our results also showed that the SUR_caudate_ and its classification performance tend to be lower than the putamen. Therefore, using an optimal VOI to calculate the SUR is necessary. Several researchers [48, 49] reported that PD patients had significantly lower DAT uptake ratios in the pallidum than healthy controls. Based on these reports, we also settled pallidum VOI, and SUR_pallidum_ of PD indicated lower than that of NC. However, it is not easy to accurately spatial normalise and delimit each region on DAT-SPECT. Therefore, we should be careful in interpreting each VOI result.

This study had some limitations. First, we employed a single-manufacturing SPECT system to exclude the influence of the differences in SPECT image quality among manufacturers. Consequently, there was an imbalance between the NC and PD groups. Therefore, it is necessary to investigate an increase in the number of patients. Second, wavelet features were not included in the radiomics features. Because SERA does not support wavelet analysis, other software should be used. Finally, we did not consider differences in the striatum laterally based on a previous report that showed lower accuracy for the asymmetry index than that of SBR and PCR [24]. As lateral differences might be useful for distinguishing between PD in the early stage and other Parkinsonism, such as progressive supranuclear palsy of the Parkinsonism subtype [50], signature construction is required.

## Conclusions

In conclusion, the radiomics signature derived from DAT-SPECT images could help distinguish between NC and PD. Furthermore, the classification performance of various classification models was improved using both radiomics signature and semi-quantitative indicators. Therefore, a radiomics signature, which includes texture information, could provide a robust diagnostic performance when used with semi-quantitative indicators.

## Data Availability

The datasets analysed during the current study are available in the PPMI repository, https://www.ppmi-info.org.

## Abbreviations

DAT-SPECT: Dopamine transporter single-photon emission computed tomography
PD: Parkinson’s disease
NC: Normal control
SUR: Striatum uptake ratio
ROC: Receiver operating characteristics
AUC: Area under the ROC curve
SBR: Specific binding ratio
3D: Three-dimensional
MNI: Montreal Neurologic Institute
AAL: Automated anatomical labelling atlas
VOI: Voxel of interest
SERA: Standardized Environment for Radiomics Analysis
SUR_caudate_: SUR of the caudate nucleus
SUR_putamen_: SUR of the putamen
SUR_pallidum_: SUR of the pallidum
CR_putamen_: Ratio of the caudate to the putamen
CR_pallidum_: Ratio of the caudate to the pallidum
LASSO: Least absolute shrinkage and selection operator
MSE: Mean square error
SVM: Support vector machine
KNN: K-nearest neighbour
LDA: Linear discriminant analysis
CI: Confidence interval
PCR: Putamen-to-caudate ratio
